# Supplementary material for: Agrin-Matrix Metalloproteinase-12 axis confers a mechanically competent microenvironment in skin wound healing
Source: Nat Commun. 2021 Nov 3;12:6349. doi: 10.1038/s41467-021-26717-7 (PMC8566503; doi:10.1038/s41467-021-26717-7)
Supplement: Supplementary file 1 — Supplementary information [file 41467_2021_26717_MOESM1_ESM.pdf]

Supplementary information

Agrin-Matrix Metalloproteinase-12 axis confers a mechanically competent microenvironment in skin wound healing

Sayan Chakraborty [1]*, Divyaleka Sampath [1], Melissa Ong Yu-Lin [1], Matthew Bilton [2,3], Cheng-Kuang Huang [2,3], Mui Hoon Nai [2,3], Kizito Njah [1], Pierre-Alexis Goy [1], Cheng-Chun Wang [1], Ernesto Guccione [1], Chwee-Teck Lim [2,3,4] and Wanjin Hong [1]*

1. Institute of Molecular and Cell Biology, Agency for Science, Technology, and Research (A*STAR), 61 Biopolis Drive, Proteos, Singapore 138673. 2. Department of Biomedical Engineering, National University of Singapore, 4 Engineering Drive 3, Singapore 117583. 3. Mechanobiology Institute, National University of Singapore, 5A Engineering Drive 1, Singapore 117411. 4. Institute for Health Innovation and Technology, National University of Singapore, 14 Medical Drive, Singapore 117599.

***Corresponding authors**

Sayan Chakraborty sayanc@imcb.a-star.edu.sg
Wanjin Hong mcbhwj@imcb.a-star.edu.sg

Supplementary figures and legends

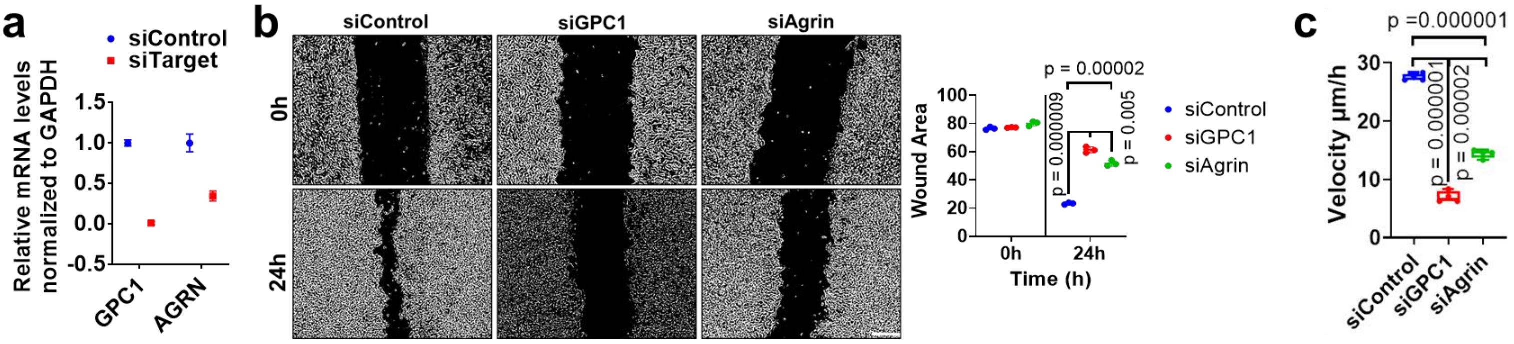

Supplementary Figure 1: Agrin and Glypican-1 play roles in keratinocyte migration post-wounding. (a) RT-PCR analysis showing the knockdown of AGRN and GPC1 in HaCaT cells. Error bars represent mean+/- s.d. from three repeats. **(b)** Control, Agrin depleted, and GPC1 knockdown cells were subjected to scratch wound assays. Representative bright-field images showing relative migration are presented at the indicated time-points. The mean non-migrated area+/- s.d. was quantified using ImageJ (n=3, Two-tailed Students 't' test, **p=0.005, ****p=0.000009, ****p=0.00002, respectively). Scale bar: 50µm. **(c)** Comparisons of migration velocities of Agrin and GPC1 knockdown HaCaTs post-wound scratch over a period of 24h (n=3, Two-tailed Students 't' test, p values given in the figure panels, Box extends from 1-99 percentile, whiskers present minimal to maximal values, central line shows median).

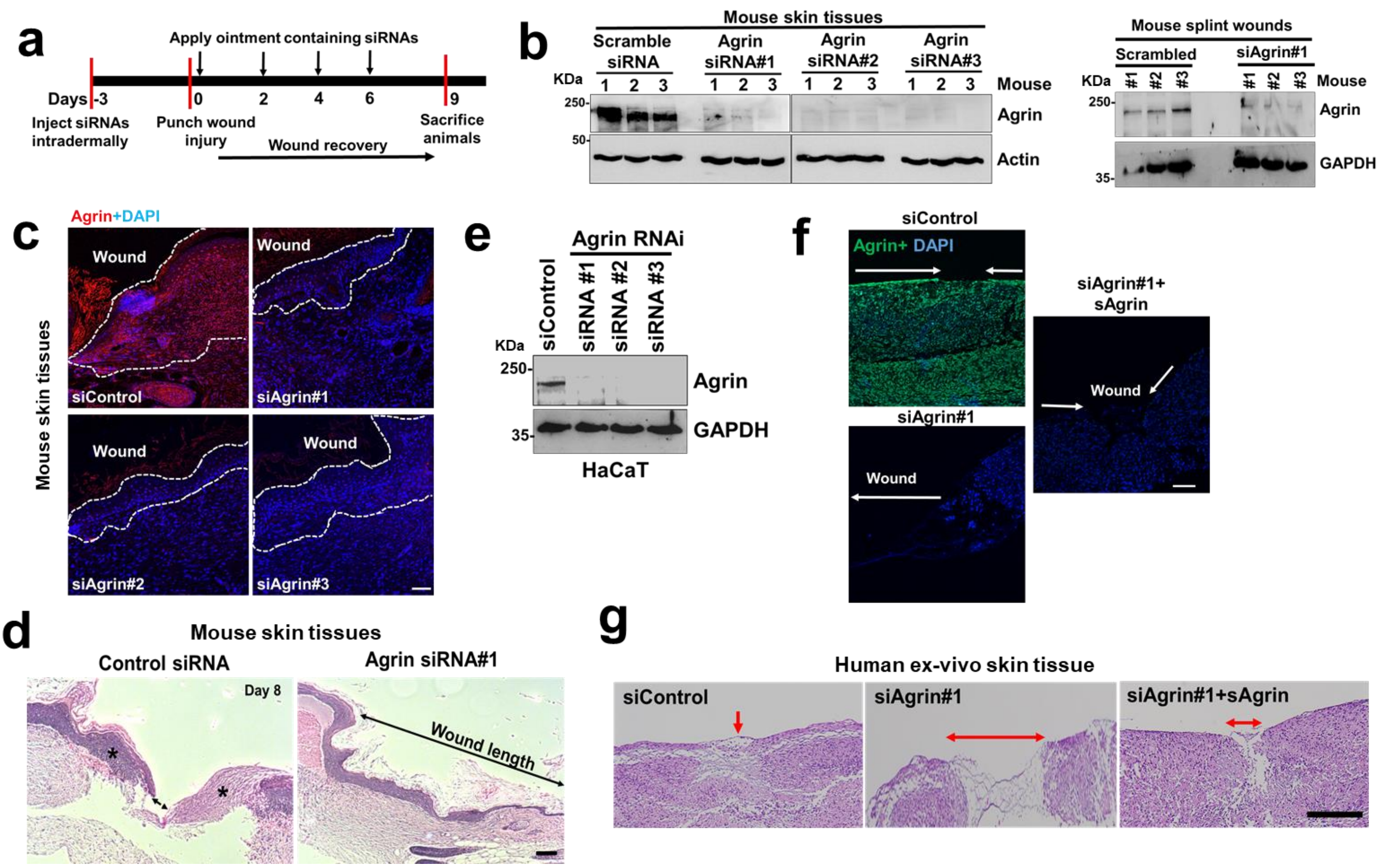

Supplementary Figure 2: Silencing Agrin delays *in vivo* wound healing. (a) Scheme showing the Agrin siRNA treatments prior to wounding and the application of siRNA containing Aquaphore ointment on wounded mice skin. Experiment repeated two times. **(b)** Western blot on mouse punch-biopsy wound lysates after 72h post-intradermal siRNA injections at day 0 of wound injury in non-splinted (left panel) and splinted (right) conditions. The mouse skin lysates were probed for Agrin. Actin or GAPDH served as loading controls (n=3 mice per group, repeated twice). **(c)** Confocal microscopy images showing Agrin immunostaining in wounded mouse skins treated with control and indicated Agrin siRNAs at day 5 post-injury in non-splinted wounds. Scale bar: 50µm. The white dashed line indicates the migrating epidermis (n= four sections from 4 mice, two experiments). **(d)** Hematoxylin and Eosin stained mouse skin sections treated with control and Agrin siRNA#1 at day 8 post-injury (n=four sections analyzed from 4 mice). The two-directional arrow refers to the uncovered wound area. The asterisk * denotes the migrating epithelial tongue covering the wound. Scale bar: 50µm. **(e)** Western blot verifying Agrin knockdown by the indicated stealth siRNAs against human Agrin in HaCaT cells. GAPDH served as a loading control. (n=2 independent repeats). **(f)** Confocal microscopy images showing Agrin immunostaining in human skin explants treated with Control and Agrin siRNA#1. One batch of Agrin siRNA treated explants were subsequently rescued with sAgrin from day 2 onwards. The represented confocal images are shown at day 8 post-wounding (n=3 explants per group, repeated twice). Nuclei are stained with DAPI. White arrows indicate the wound area left uncovered. Scale bar: 50µm. **(g)** Representative Hematoxylin and Eosin stained sections of human full-thickness skin explants treated with either 75nM Control or Agrin siRNA#1 at day 8 post-injury. The third panel consists of skin explant sections that were treated with Agrin siRNA#1 ointment on day 0, but subsequently received ointments that additionally contained sAgrin (20µg) on days 2, 4 and 6, respectively. The red arrow

indicates the migrating epithelial tongue covering the wound area in control skin sections. The double-direction red arrow refers to the uncovered wound areas (n=3 human skin explants per group, repeated twice). Scale bar: 500µm.

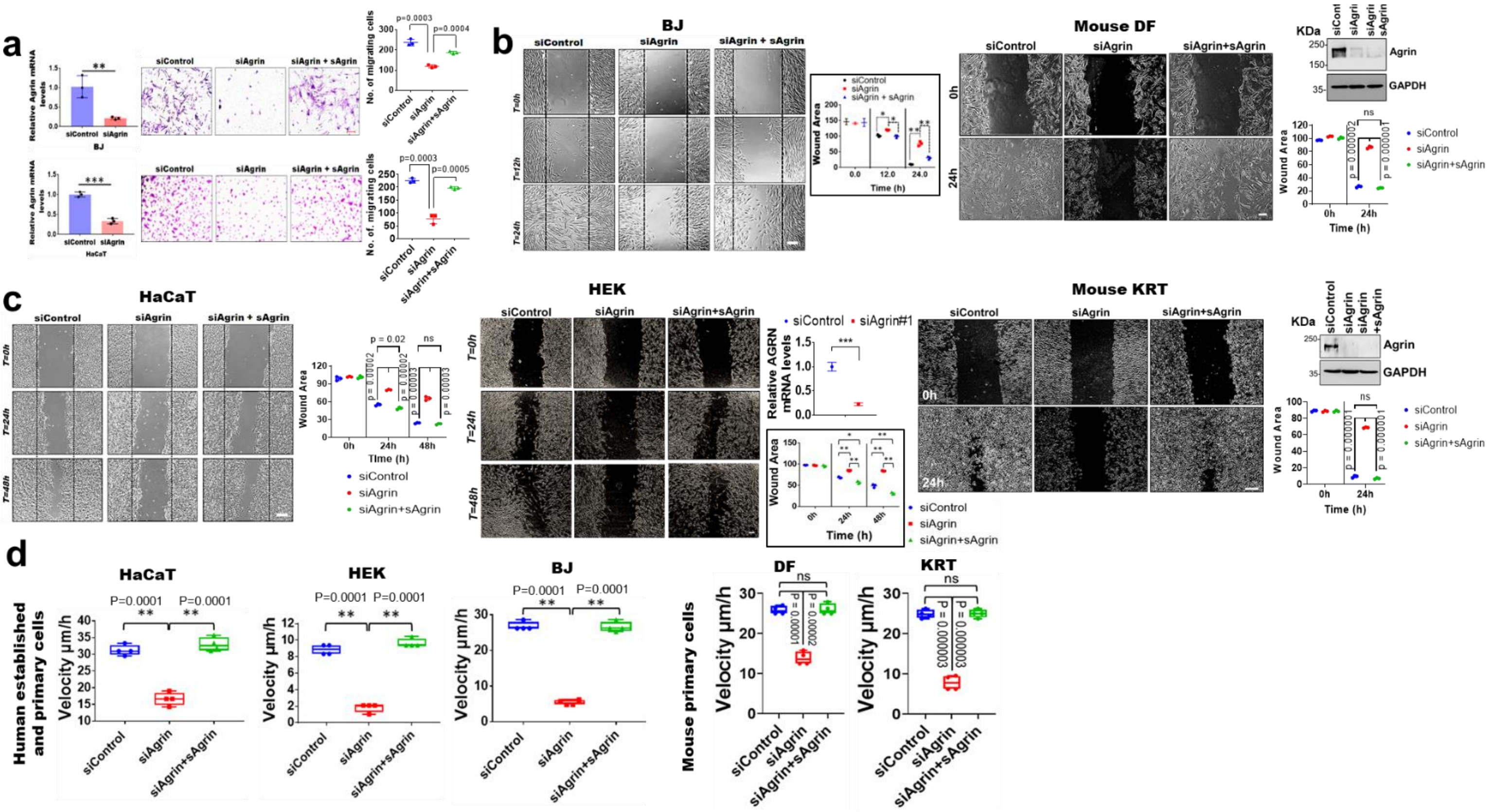

Supplementary Figure 3: Agrin knockdown hampers 2D migration of keratinocytes and dermal fibroblasts. (a) RT-PCR analysis for Agrin gene expression in BJ and HaCaT cells treated with either control or Agrin siRNA. Data presented as mean+/- s.d. in three technical repeats (Students 't' test, Two-tailed, **p=0.002, ***p=0.0005, respectively). These knockdown cells were subsequently used for transwell migration assays. Migration was analyzed for a batch of Agrin depleted cells in response to sAgrin (10µg/ml) in the bottom chambers as a chemoattractant (third panels). Migrating were quantified using ImageJ and represented as mean+/- s.d. (n=3, Two-tailed Students 't' test, p=0.0004, 0.0003, and 0.0005, respectively). Scale bar: 20µm. **(b)** Control, Agrin depleted, and Agrin knockdown BJ or primary mouse dermal fibroblasts (DF) cells pre-treated with 10µg/ml sAgrin for 18h were subjected to scratch assays. Representative bright-field images showing relative migration are presented at the indicated time-points. The mean non-migrated area+/- s.d. was quantified using ImageJ (n=3, Two-tailed Students 't' test, *p=0.05, **p=0.004, ***p=0.0005, ****p=0.000002, ****p=0.000001, respectively). Scale bar: 20µm. A Western blot verifying the knockdown of Agrin in mouse DFs is also shown with GAPDH as loading control. **(c)** Scratch assay in control, Agrin depleted, and Agrin knockdown indicated keratinocytes pre-treated with 10µg/ml sAgrin for 18h. Representative brightfield images are shown. Scale bar: 20µm. RT-PCR analysis verifying Agrin expression in HEK cells is presented as mean+/- s.d (middle panel, two repeats), while a Western blot confirming Agrin knockdown is presented for primary mouse keratinocytes (rightmost panel). Results are quantified as in panel (b) (n=3, Two-tailed Students 't' test, ****p=0.000001, **p=0.005, *p=0.02, respectively, all other p values are represented in the panels). **(d)** Comparisons of migration velocities of indicated cells treated as in panels (b and c) at 15 h post-wounding (n=4 independent migration experiments, Two-tailed Students 't' test, p values indicated in the figure, Box extends from 1-99 percentile, whiskers represent minimal to maximal values, central line shows median values).

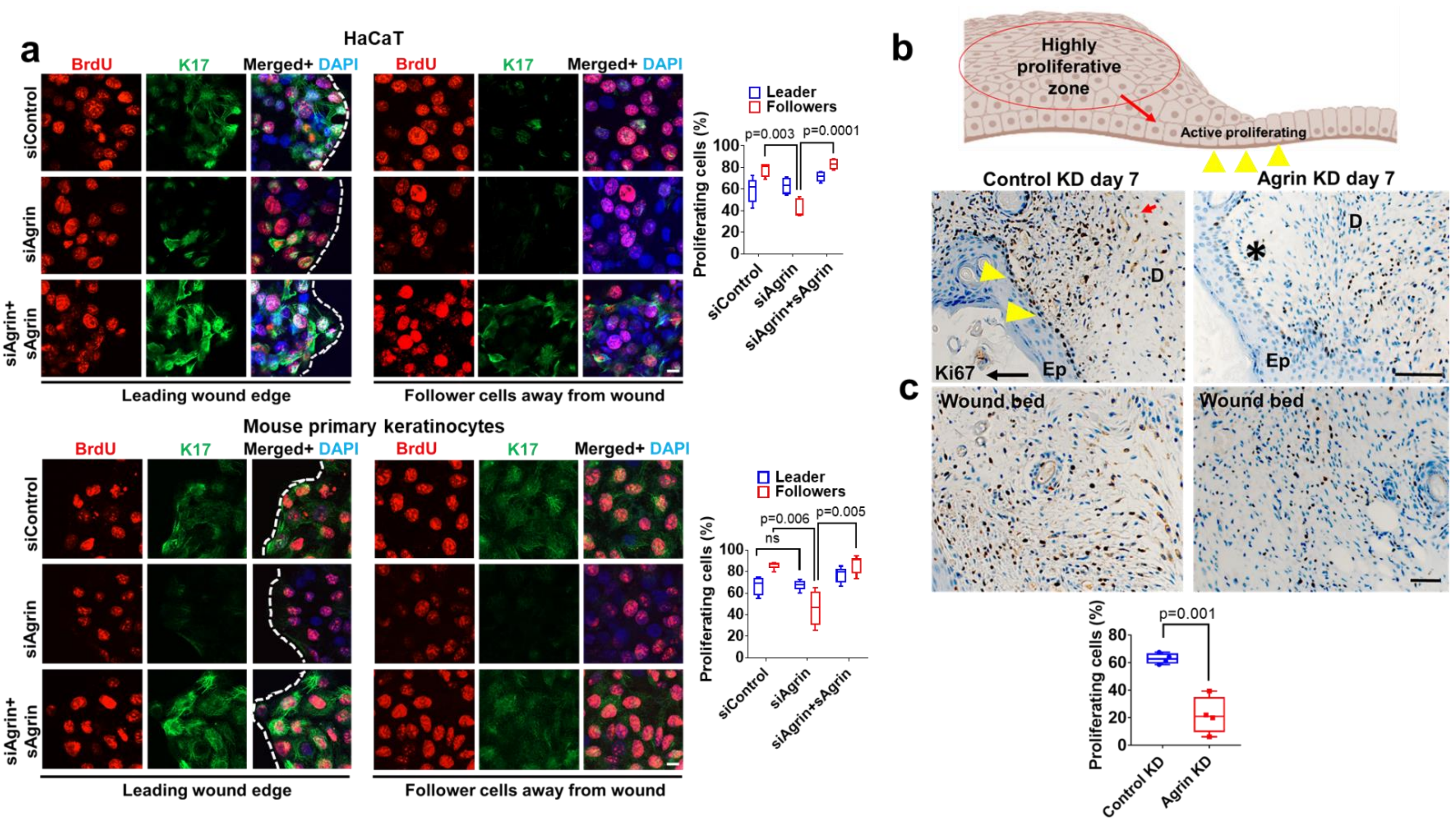

Supplementary Figure 4: Agrin impacts keratinocyte proliferation following wounding. (a) Control, Agrin depleted indicated keratinocytes and those rescued with 10µg/ml sAgrin for 18h were labelled with 20µM BrdU for 12 hours, before proceeding with scratch assays. The cells were fixed at 4 hours post-scratching and stained with an Anti-BrdU and K17 antibodies. Nuclei were counterstained with DAPI. Representative confocal images at the wound edge (leader) and at-least 200µm away from the wound margin (followers) are shown. The relative percentage of proliferating cells are shown for each condition presented in box-whisker plots (n=5 images analyzed for each conditions from three experiments, Two-tailed Students 't' test, ** p=0.006, *p-0.003, p=0.005, ***p=0.0001, respectively, Box extends from 1-99 percentile, Whiskers range from minimum to maximum values, central line shows median). Scale bar: 10µm. **(b-c)** Schematic showing the states of proliferation at the migrating wound margins. Immunohistological sections stained for Ki67 in control and Agrin depleted wound edges and beds at day 7 post-wounding. Yellow arrows denote the base of epidermis (Ep), while red arrows represent the wound margin. Asterisk (*) denotes the loss of Ki67 proliferation at the wound margins. Scale bar: 100µm. The percentage of proliferating cells was quantified using Image J (n=4 sections analyzed from two individual mice, Two-tailed Students 't' tests, **p=0.001, Box extends from 1-99 percentile, Whiskers range from minimum to maximum values, central line shows median).

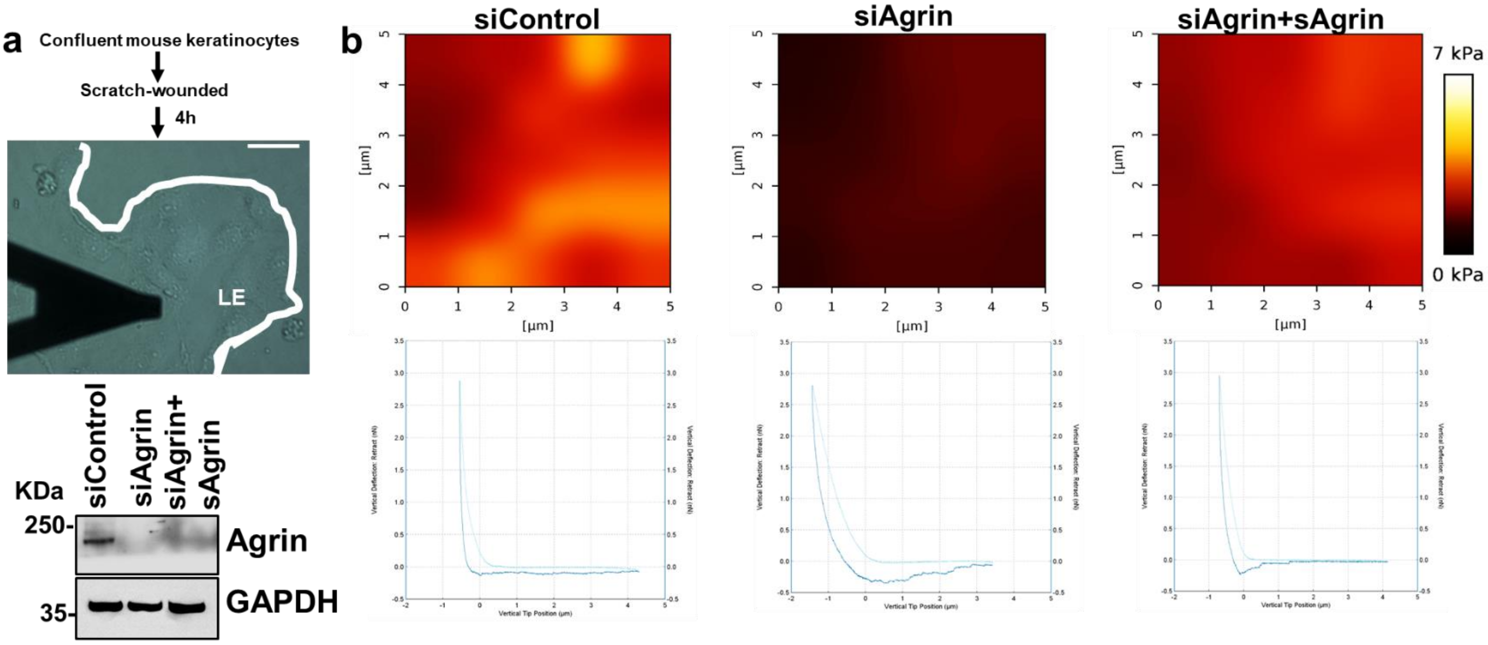

Supplementary Figure 5: Agrin attributes stiffness to migrating keratinocytes. (a) Control, Agrin depleted, and Agrin knockdown primary mouse keratinocytes cells pre-treated with 10µg/ml sAgrin for 18h were subjected to scratch assays. At 4h post-scratching, the cells at the leading edge (LE) (white line) were analyzed by AFM. Representative Western blot verifying Agrin knockdown is shown with GAPDH as a loading control (n=3 independent experiments). Scale bar: 10µm. **(b)** AFM stiffness map and force curves of indicated conditions are shown. Force scale represents 0-7KPa. Each experiment was repeated three times.

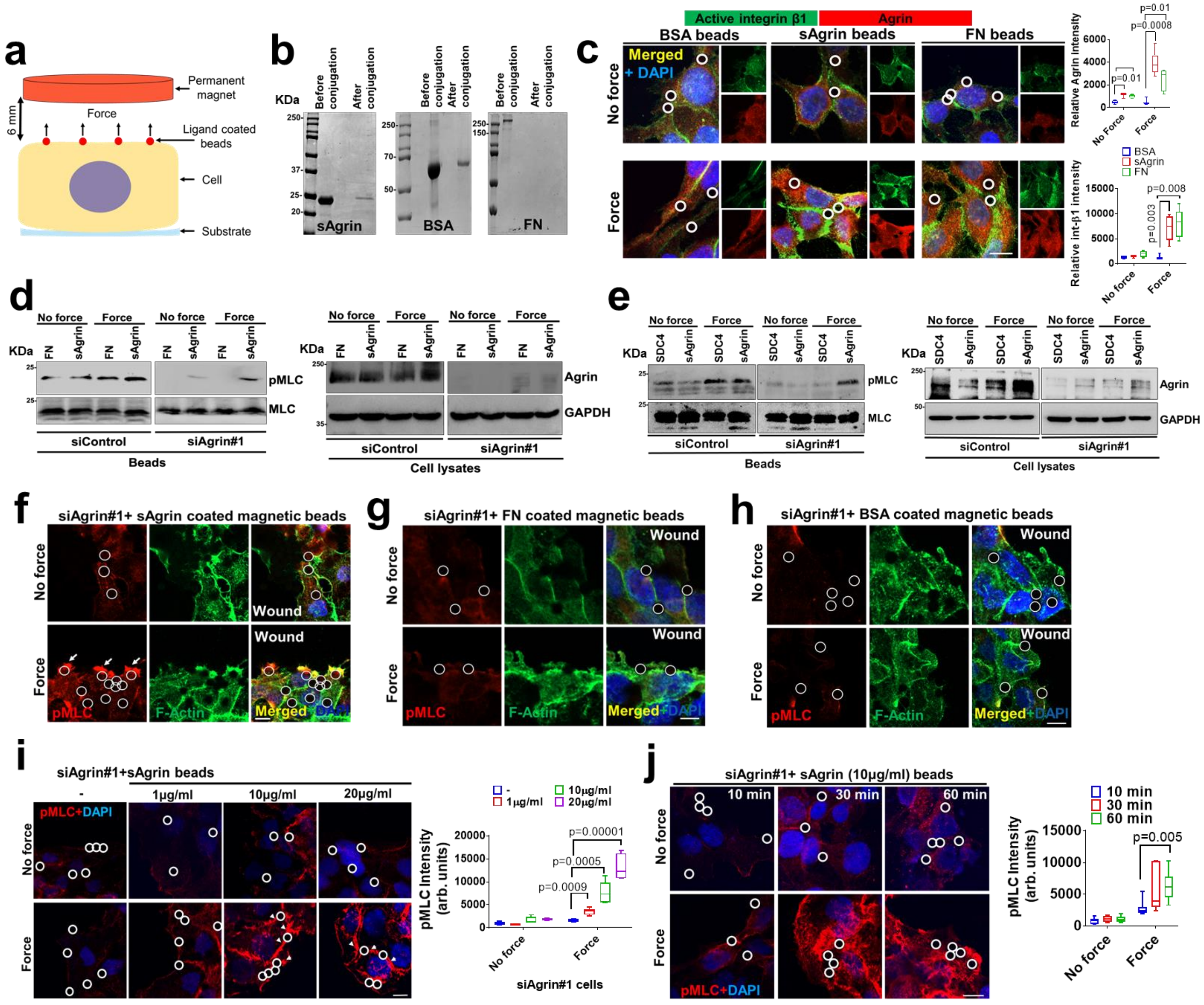

Supplementary Figure 6: Force recognition by Agrin to wounded keratinocytes engages acto-myosin. (a) Set-up of magnetic force transmission to wounded keratinocytes. Briefly, keratinocytes (HaCaT) were coated with pre-conjugated magnetic beads with either control or sAgrin for 30 min at 4°C. The cells were subsequently scratched and allowed to migrate at 37°C either under standard culture conditions or under a permanent magnet placed 6 mm above the cells that exerted a force of 200pN for 30 min. **(b)** SDS-PAGE gel showing the respective conjugation of 20 µg ligands- sAgrin, Bovine Serum Albumin (BSA) and Fibronectin (FN) to magnetic beads. The respective free proteins (probed in the supernatants before incubation with beads) are diminished in the bead-free supernatants after covalent conjugation. Experiment was repeated three times. **(c)** HaCaT cells coated with BSA, sAgrin or FN beads for 30 min, were allowed to migrate under standard conditions or a permanent force for an additional 30 min. The cells were subsequently fixed and stained for active Integrin β1 (HUTS-4 clone) and Agrin. Nuclei were counterstained with DAPI. The white circles denote the bead location. Scale bar: 10µm. The mean intensity at an area of 200µ^2 around each bead was quantified from three experiments (n=10 cells per group, Holm-Sidak Multiple t tests, p values presented in panels, Box extends from 1-99 percentile, whiskers represent minimum to maximum values, central

line depicts median). **(d-e)** Control and Agrin siRNA#1 treated HEK cells were subjected to force transduction as in (b) with sAgrin and FN (d) or syndecan 4 beads (e) after 72h post-siRNA treatments. The beads adhered proteins were magnetically separated and were resolved in a Western blot analysis probing for pMLC and total MLC. Respective cell lysates were probed for Agrin and GAPDH served as a loading control (n=2 independent experiments). **(f-h)** Representative confocal images of Agrin depleted HEK cells being probed with sAgrin (f), FN (g) or BSA (h) coated magnetic beads under standard conditions (no force) or application of permanent force. Cells were fixed and immunostained for pMLC and F-actin, respectively. DAPI marked the nuclei. White circles represented the beads (n=3 independent experiments). Scale bar: 10µm. **(i)** Agrin depleted HaCaT cells were exposed with increased concentrations of sAgrin-coated magnetic beads for 30 min. Following wounding, the cells were allowed to migrate for 30 min under no force or permanent force applications and subsequently fixed and stained for pMLC. The nuclei were stained with DAPI. The block arrows represent enhanced pMLC recruitment near beads. The pMLC intensity presented as mean+/- s.d. in a $200\mu^2$ area around the beads was quantified from three experiments (n=10 cells, Holm-Sidak Multiple t tests, p values presented in the panel, box indicates 1-99 percentile, whiskers represent minimum to maximum values, central line show median). Scale bar: 10µm. **(j)** Agrin depleted HaCaT were treated with sAgrin beads and allowed to migrate under sustained force applications for the indicated time-points. The resultant cells were quantified as in (i) (n=10 cells, repeated twice, Holm-Sidak Multiple t tests, p values presented in the panel, Box extends from 1-99 percentile and whiskers represent minimum to maximal values, central line represents median values, respectively).

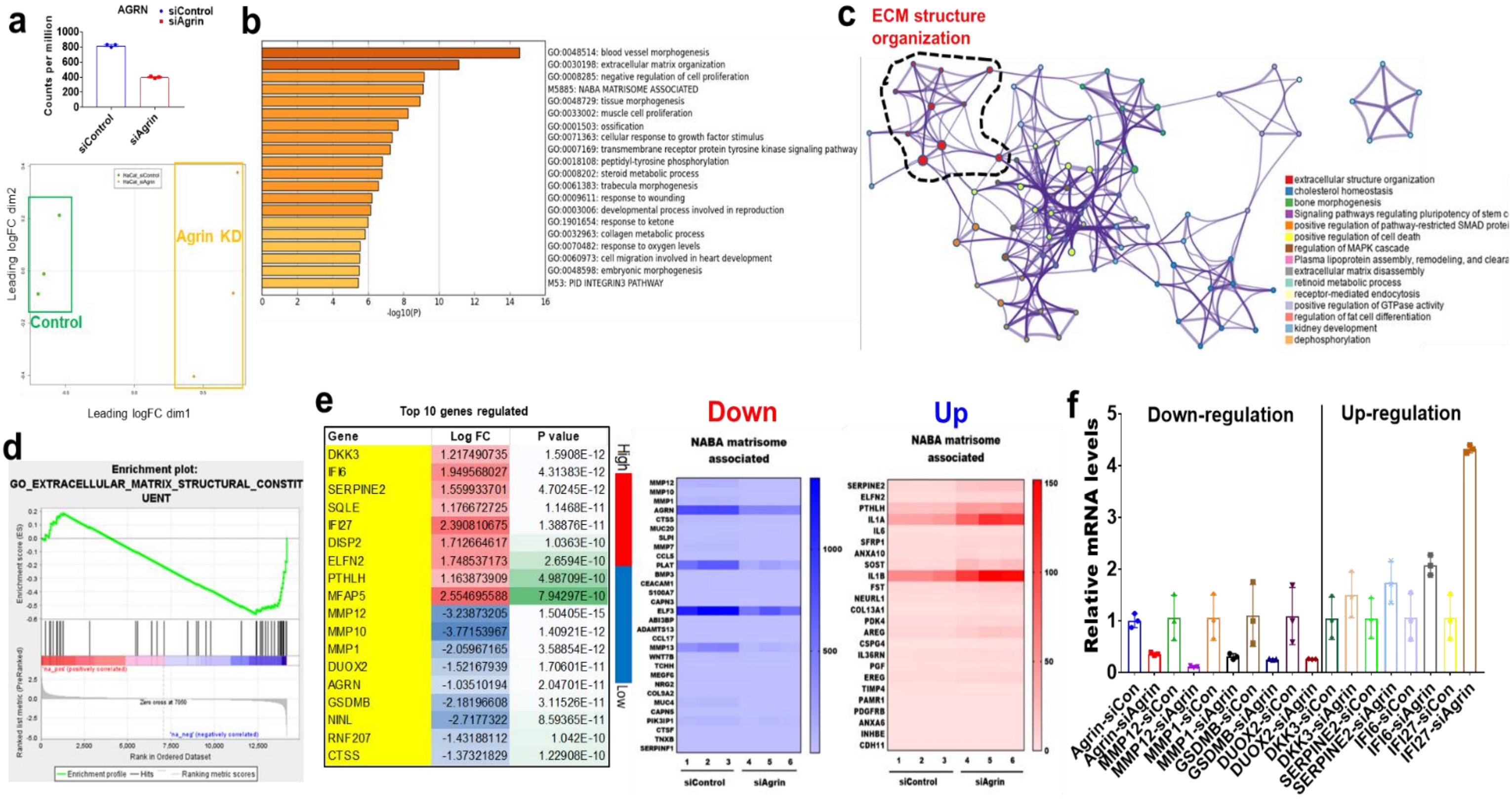

Supplementary Figure 7: Transcriptome analysis of Agrin depleted keratinocytes. (a) RNA-Seq revealing a loss of Agrin gene expression (n=3 replicates). MDS plot of bulk population of RNA-Seq from siControl (green dots) and siAgrin (yellow) HaCaTs (n=3 samples). **(b)** Gene ontology (GO) analysis in Agrin knockdown HaCaT cells showing the most significantly down-regulated gene clusters. **(c)** Cytoscape network analysis of RNA-Seq data from Agrin depleted HaCaT cells showing that the highly regulated cluster of gene(s) belongs to the ECM structure organization cluster (red nodes). The size of the nodes is dependent on the significance (p-values), while the distance between the nodes denotes the degree of clustering across the panel of genes. **(d)** GSEA analysis upon Agrin knockdown reveals that gene sets belonging to the ECM structural constituents are highly down-regulated. **(e)** Chart showing top ten up- (red) and down-regulated (blue) genes by Agrin depletion. Differentially expressed genes were obtained by quasi-likelihood (QL) F-test (edgeR). Up- and down-regulated gene clusters in the category of NABA matrisome (associated with ECM) upon Agrin depletion in HaCaT cells (p-values obtained from Gene Ontology analysis). **(f)** RT-PCR analysis validating a selected set of significantly up-and down-regulated genes in HaCaT cells. Error bars represent mean+/- s.d. from three independent experiments.

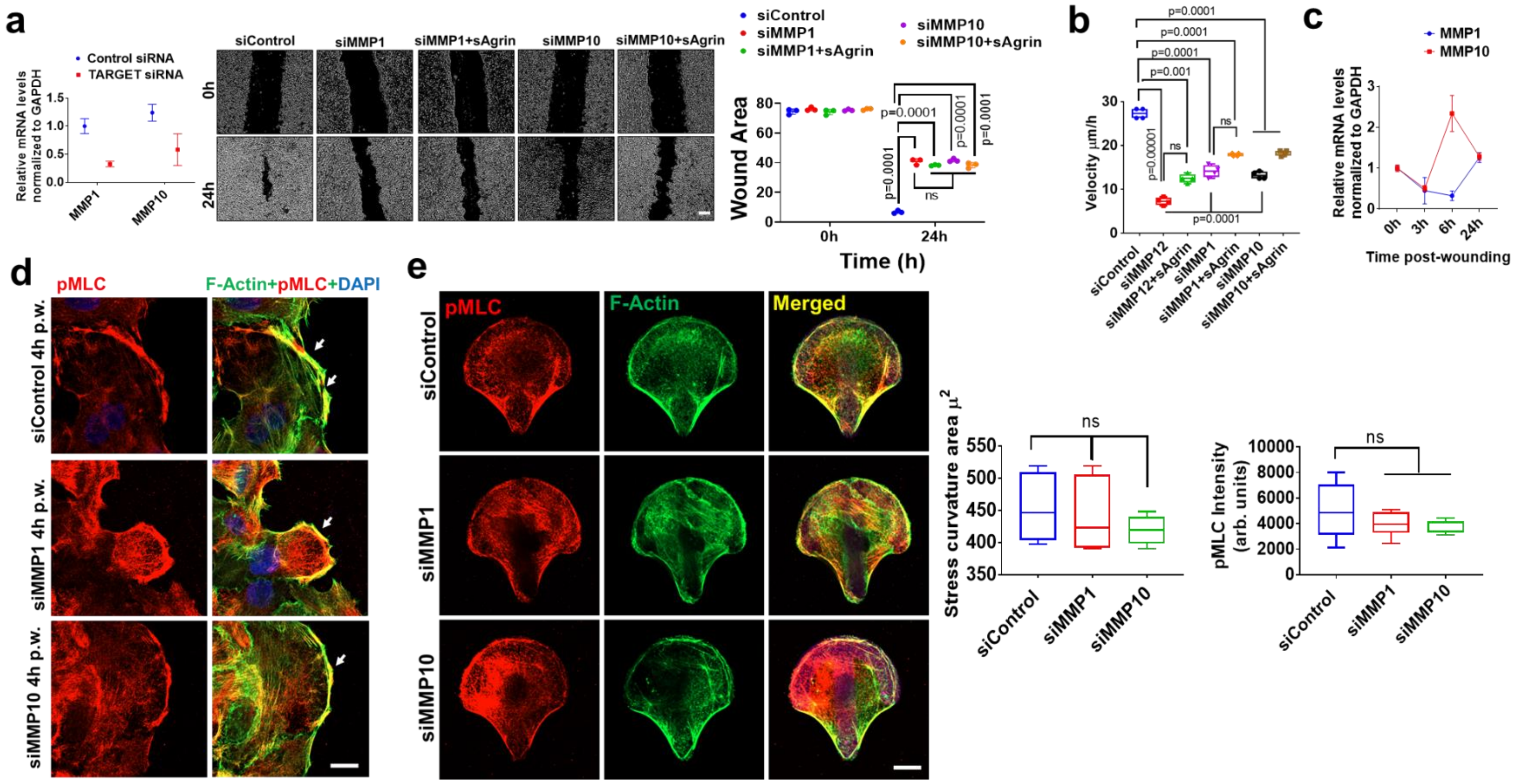

Supplementary Figure 8: Depletion of MMP1 and MMP10 impairs wound healing without affecting cellular mechanotension. (a) RT-PCR analysis confirming the suppression of MMP1 and MMP10 in HaCaT cells (Error bars represent mean+/- s.d., from two experiments). Control, MMP1 and 10 depleted HaCaTs and those pre-treated with 10µg/ml sAgrin for 18h were subjected to scratch assays. Representative bright-field images showing relative migration are presented at the indicated time-points. The mean non-migrated area+/- s.d. was quantified using ImageJ (n=3 independent experiments, Two-tailed Students ‘t’ test, p values indicated in the figure). Scale bar: 50µm. **(b)** Box-whisker plots showing migration velocities of indicated knockdown cells either alone or treated with 10µg/ml sAgrin at 15h post-wounding (n=4, Two-tailed Students ‘t’ tests, ****p=0.00001, ***p=0.0001, **p=0.001, respectively, Box- 1-99 percentile, whiskers-minimum to maximum, central line-median). **(c)** RT-PCR analysis showing mRNA expression of MMP1 and 10 at indicated time-points post-scratching in HaCaT cells. Error bars represent mean+/- s.d. from two experiments performed in three technical repeats. **(d)** Control, MMP1 and MMP10 depleted HaCaT cells were scratched and allowed to migrate for 4h. The cells were subsequently fixed and immunostained for pMLC and F-actin. Representative confocal images are shown. Nuclei were counter stained with DAPI. White arrows indicate acto-myosin cables at the leading edge (n=3 independent experiments). Scale bar: 10µm. **(e)** Control, MMP1 and MMP10 depleted HaCaT cells were plated on large crossbow patterns for 6h. The cells were subsequently fixed and stained for pMLC and F-actin, with DAPI marking the cell nuclei. White arrows refer to pMLC enriched transverse arcs. Box-whisker plots of stress curvature enriched with pMLC from three independent experiments (n=6 cells per group, repeated twice, Two-tailed Students ‘t’ test, p=0.5 and p=0.16, respectively, Box extends from 1-99 percentile, whisker represent minimum to maximum values, central line represents median values).

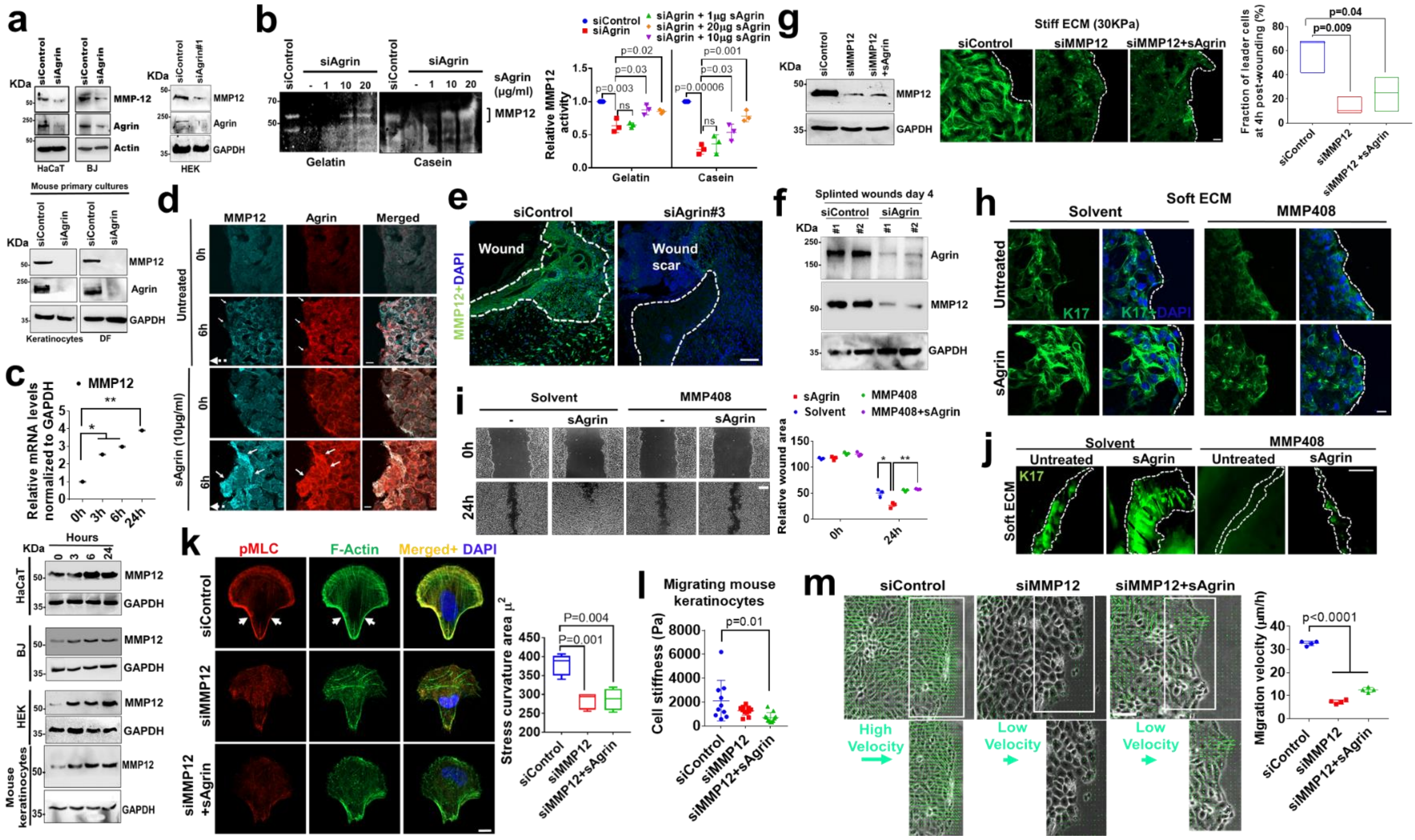

Supplementary Figure 9: Agrin-regulated MMP12 upgrades cellular mechanics and collective fluidic migration. (a) Cell lysates of control and Agrin siRNA treated cells were analyzed by Western blot for MMP12 and Agrin expression. Actin and GAPDH served as loading controls, respectively, n=3 independent experiments. **(b)** Gelatin and casein zymography detecting the catalytic activity of MMP12 in control and Agrin depleted HaCaT cell supernatants. The cell supernatants of Control, Agrin siRNA treated alone, or those treated with an increasing dose of sAgrin for 24h were analyzed for MMP12 catalytic activity by gelatin (left) or casein (right) in-gel digestion. The appearance of MMP12 bands (~54KDa) are a result of gelatin digestion by MMP12. Representative image from one of three independent experiments is shown. The relative MMP12 band intensity was quantified using ImageJ (n=3, Two-tailed Students 't' test, Holm-Sidak Multiple t tests, p values presented in figure). **(c)** RT-PCR analysis detecting MMP12 mRNA in migrating HaCaTs at 0h and 24h post-wound injury. Data presented as mean+/- s.d. Two-tailed Students t test, *p=0.01, **p=0.002, n=3 replicates, respectively. Indicated cells were wounded and allowed to migrate. At the indicated time-points, cell lysates from the migrated cells were collected and analyzed by Western blot for MMP12. GAPDH served as loading controls (n=3 independent experiments). **(d)** Confluent HaCaT cells were either left untreated or pre-treated with sAgrin (10μg/ml) for 18h, before scratching them and allowing migration for 6h. At the indicated time-points, cells were fixed and immunostained for MMP12 and Agrin, respectively. Representative confocal images are shown. White arrows indicate leader cells at the migrating edges (n=2 independent repeats). Scale bar: 10μm. White block arrow indicates the direction of migration. **(e)** Representative confocal microscopy images of control and Agrin depleted mouse skin sections showing MMP12 expression at the wound edges. The White dashed line indicates the migrating epithelial tongue. Nuclei are counterstained with DAPI. Scale bar: 50μm (n=3 sections from three mice analyzed per condition). **(f)** Western blot analysis showing MMP12 levels on control and Agrin depleted mouse skin tissues at day 4 under

splinted conditions. GAPDH was used as a loading control (n=2 mouse wounds per group, two repeats). **(g)** Control and Agrin depleted HaCaT cells were cultured on stiff substrates. One batch of Agrin depleted cells were cultured on stiff substrates that contained 20μg/ml sAgrin. Western blot confirming the loss of MMP12 expression in HaCaT cells. GAPDH was used as a loading control. Upon confluency, the cells were scratched and allowed to migrate for 4h. The cells were then fixed and immunostained for K17. The dashed line denotes the migrating front. The K17 intensity in the leader cells was quantified from two independent experiments (n=20 cells, Two-tailed Students 't' test, *p=0.009 and 0.04, respectively, Floating bars show represent 1-99 percentile values, central line at median). **(h-i)** HaCaT cells were either untreated or pre-treated with 10μg/ml sAgrin for 18h and cultured on soft 0.8KPa substrates (h) or plastic dishes (i). Subsequently, the cells were pre-treated with solvent (DMSO) or 5nM MMP408 inhibitor for 2h, before scratching them and allowing them to migrate for an additional 4h in the presence of solvent or inhibitor. Confocal images showing K17 and nuclei stained with DAPI (h) are represented. The White dashed line presents the migrating front. Scale bar: 10μm (h) and 50μm (i), respectively. Bright-field microscope images at indicated times show the migrating cells (i). Three independent repeats were done (h-i). The uncovered wound area was quantified as mean+/- s.d. (n=3, Two-tailed Students 't' test, *p<0.05, **p<0.005, respectively). Scale bar: 50μm. **(j)** Confocal images revealing the K17 immunostained outgrowths from mouse skin explants cultured on soft substrates (0.8KPa) alone or those supplemented with sAgrin in the presence of DMSO or 5nM MMP408 for 5 days (n=2 independent experiments, with 3 skin explants per group). The white dashed line represents the outgrowth area. Scale bar: 50μm. **(k)** Control and MMP12 depleted HaCaT cells were plated on large FN-coated micropatterns for 4-6h. Besides, a batch of MMP12 depleted cells were plated on FN patterns containing 20μg/ml sAgrin for 4-6h. The cells were subsequently fixed and stained for pMLC and F-actin, with DAPI marking the cell nuclei. White arrows refer to pMLC enriched transverse arcs. The stress curvature associated are enriched with pMLC was quantified from three independent experiments (n=15 cells per group, Two-tailed Students 't' test, **p=0.001, 0.004, Box extends from 1-99 percentile, whiskers show minimum to maximum values, central line depicts median). Scale bar: 10μm. **(l)** AFM computation of cell stiffness in control, MMP12 depleted and MMP12 knockdown primary mouse keratinocytes treated with sAgrin (n=10 cells analyzed from three independent experiments, Two-tailed Students 't' test, *p=0.01) **(m)** PIV analysis for fluidic velocity during collective migration of control, MMP12 knockdown cells and those treated with sAgrin. Green arrows represent the velocity vectors. Scale bar: 100μm. The migration velocities are quantified as mean+/- s.d. from four independent experiments (Two-tailed Students't' test, ***p=0.000001).

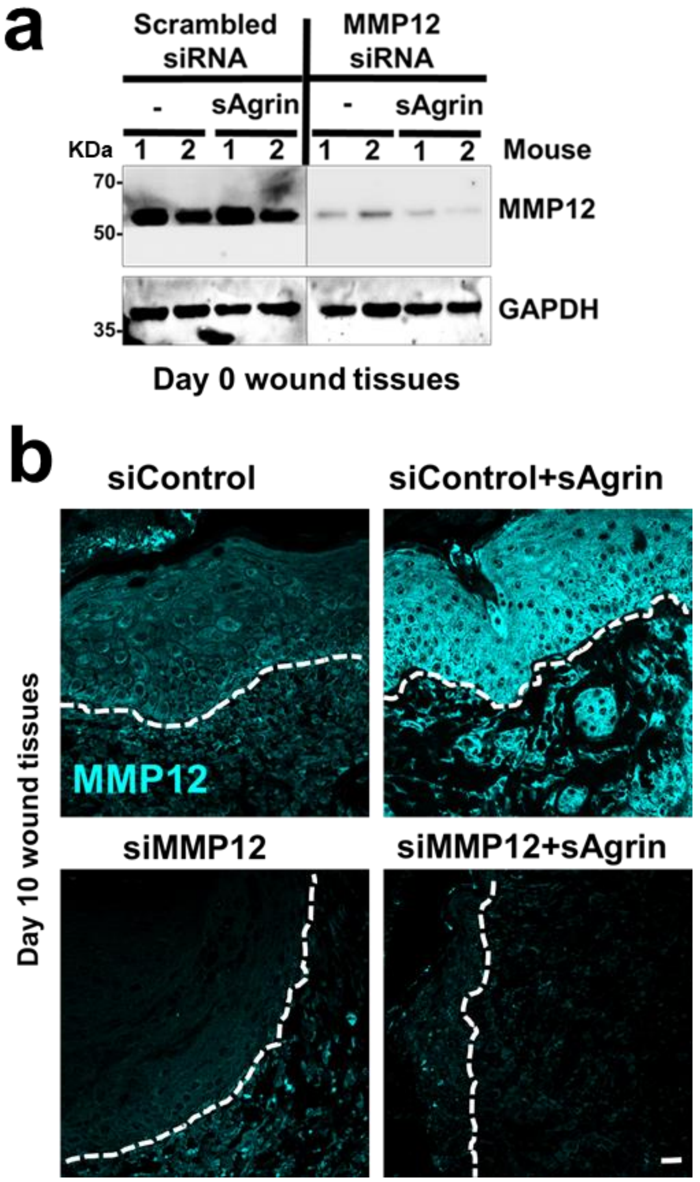

Supplementary Figure 10: Knockdown of MMP12 in the mouse skin. (a) Mouse skin were treated with control or MMP12 specific siRNAs. Three days later skin tissues were either collected in the presence or absence of 200 µg sAgrin, and Western blotted for MMP12 levels. GAPDH served as loading controls. Two independent mouse wounds were analyzed for two independent experiments. **(b)** Confocal imaging of mouse skin tissues treated same as in (a) at day 10 post-wound injury showing MMP12 expression. White dashed line represents the epidermal and dermal boundaries. Three sections from two different mouse were analyzed for each condition from two experiments. Scale bar: 10µm.

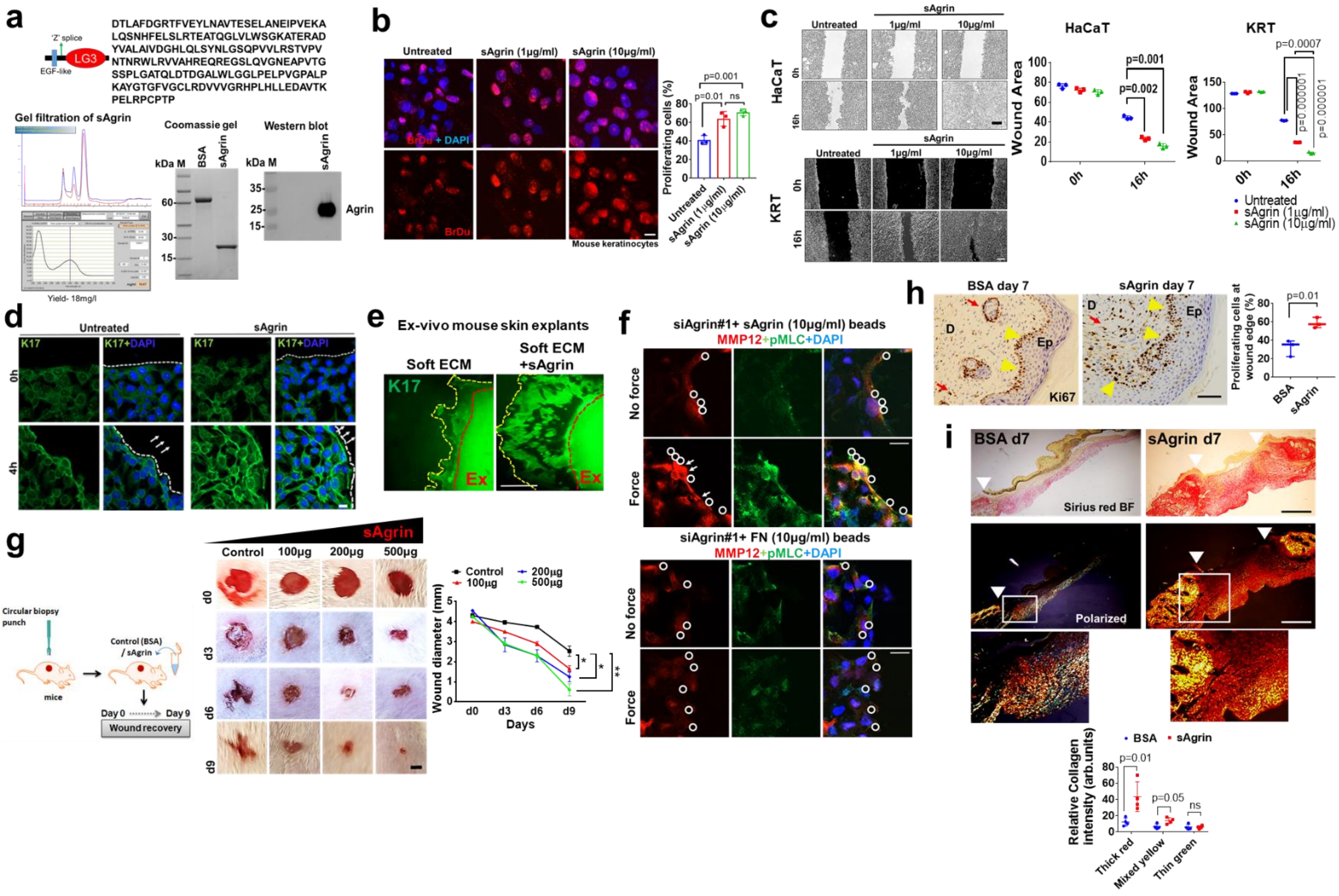

Supplementary Figure 11: sAgrin as a bio-additive skin wound-healing material. **(a)** The protein sequence of the C-terminal Agrin fragment used as bio-additive. Gel-filtration profile of purified sAgrin. The recombinant sAgrin were tested by Coomassie stained gel and Western blot using an Agrin antibody. Three independent experiments were performed. **(b)** BrdU proliferation assay of mouse keratinocytes cells treated with increasing concentrations of sAgrin for the indicated days (n=3 experiments, Two-tailed Students't' test, **p=0.001 and *p=0.01, respectively). Scale bar: 10μm. **(c)** HaCaT and mouse keratinocytes treated with increasing doses of sAgrin were subjected to scratch assay. Representative bright-field images are shown at indicated times post-scratching. Scale bar: 20μm. The resulting uncovered wound area presented as mean+/- s.d. was quantified using ImageJ (n=3, Two-tailed Students 't' test, p values indicated). **(d)** Untreated or sAgrin (10μg/ml) HaCaT cells were scratched and allowed to migrate for 4h. Subsequently, the cells were fixed and immunostained for K17 and nuclei was stained with DAPI. Representative confocal images are presented. White arrows point towards the direction of migration. The white dashed line represents the migrating front (n=2 independent experiments). Scale bar: 10μm. **(e)** Confocal images showing K17 staining from mouse skin explants cultured for 5 days on soft substrates alone or those supplemented with sAgrin (20μg/ml). 'Ex' refers to explant and the red line represents the border of explant. The region between the red and yellow lines presents the outgrowth areas, respectively. Scale bar: 50μm. The experiment was done three times with three explants per group. **(f)** Agrin depleted HaCaTs were treated with sAgrin or FN coated beads for 30 min before wounding them. The cells were either left alone or placed under magnetic force and allowed to migrate for 30 min

before they were fixed and immunostained for MMP12 and pMLC. Nuclei were counterstained with DAPI. Representative confocal images are shown (n=2 experimental replicates). Scale bar: 10μm. White circles denote the beads. **(g)** Vaseline based ointments containing either 200 μg BSA (control) or increasing amounts of sAgrin were applied topically to punch-wounds in mouse skin every 2-3 days. The representative photographs of the treated mice wound regions are shown on indicated days. Scale bar: 1mm. The relative diameter of wounds presented as mean+/- s.e.m. was measured on indicated days from one experiment (n=3 mice per group, Two-way ANOVA, *p=0.04, **p=0.004, respectively). **(h)** Ki67 stained immunohistology sections of BSA or sAgrin treated mouse skin at day 7 post-injury. The degree of proliferating cells were quantified using ImageJ (Error bars represent mean +/- s.d. from n=3 sections analyzed from three independent mice wounds, Two-tailed Students 't' tests, *p=0.01). Yellow arrows represent the proliferative base of epidermis while red arrows refer to the wound margins, respectively. **(i)** Picrosirius red stained images of BSA and sAgrin treated mouse skin sections (non-splinted model) at day 7 post-wounding. Representative bright-field and polarized views are presented. Scale bar: 100μm. White arrows represent the region of unhealed area. The quantification of individual types of collagen fibers were presented as mean+/- s.d. (n=4 sections analyzed per group from three mice, Two-tailed Students 't' test, *p=0.01 and 0.05, respectively).

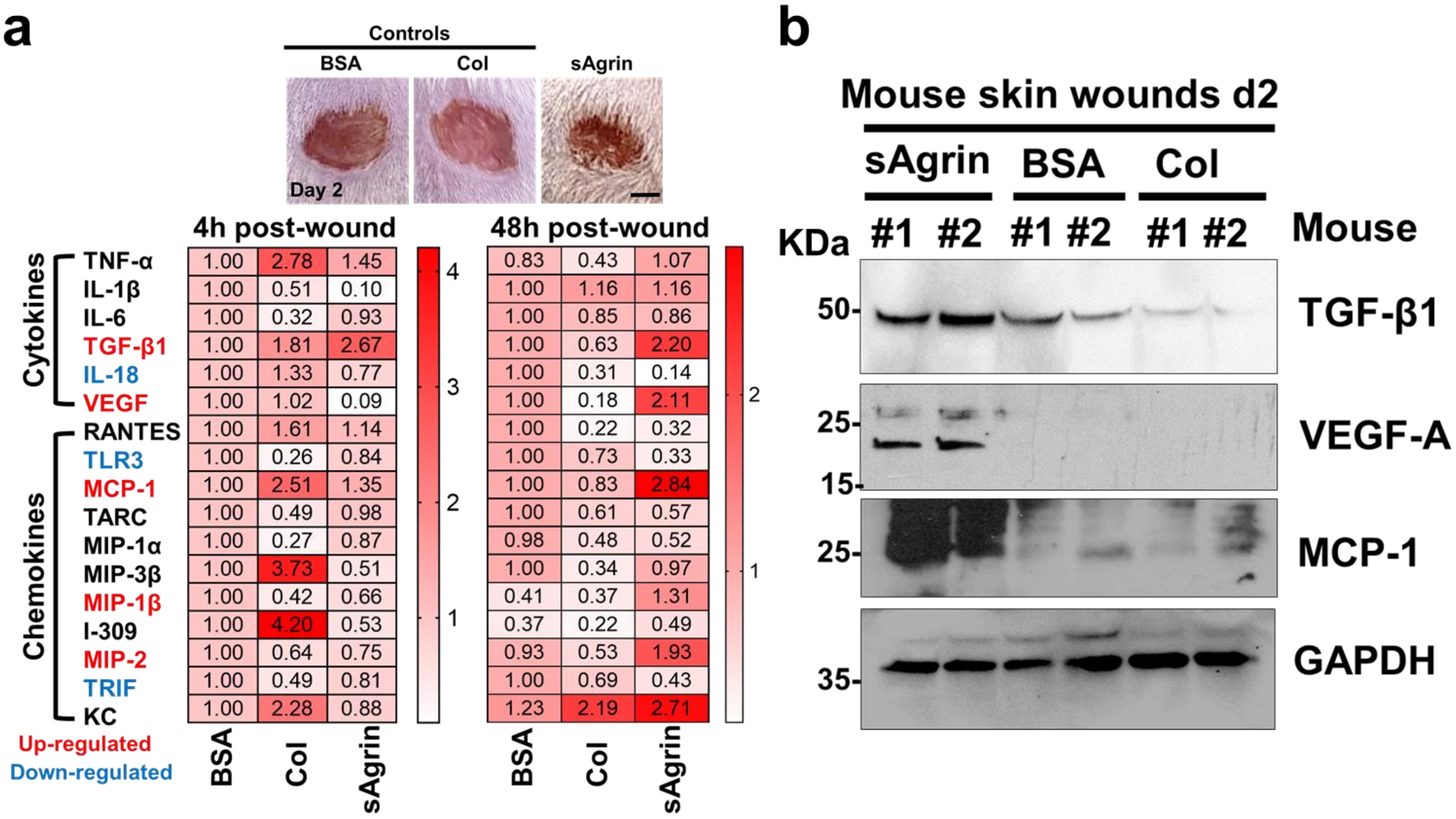

Up-regulated pro-inflammatory genes	BSA vs Col	BSA vs sAgrin	Col vs sAgrin
TGF-β1	0.94	0.01	0.01
VEGF	0.01	0.005	0.0001
MCP-1	0.81	0.0006	0.03
MIP-1ß	0.32	0.01	0.01
MIP-2	0.22	0.01	0.008

Supplementary Figure 12: Profile of pro-inflammatory proteins induced by sAgrin during early phases of wound healing. (a) Heatmap showing the mRNA expression(s) of commonly induced cytokines and chemokines at 4h and 48h post-wounding in mouse skin that received BSA, Collagen or sAgrin treatments. The photographs of a representative wound for each condition is shown above. The p values for the selected group of significant genes are represented in tabular form (n=3 mice per group, Holm-Sidak method Multiple t tests, performed in 3 technical repeats). **(b)** Western blot analysis in the mouse skin treated as in (a) showing the protein expression of selected proteins. GAPDH served as a loading control (n=2 independent mice wounds were analyzed for each group, experiment was repeated twice).

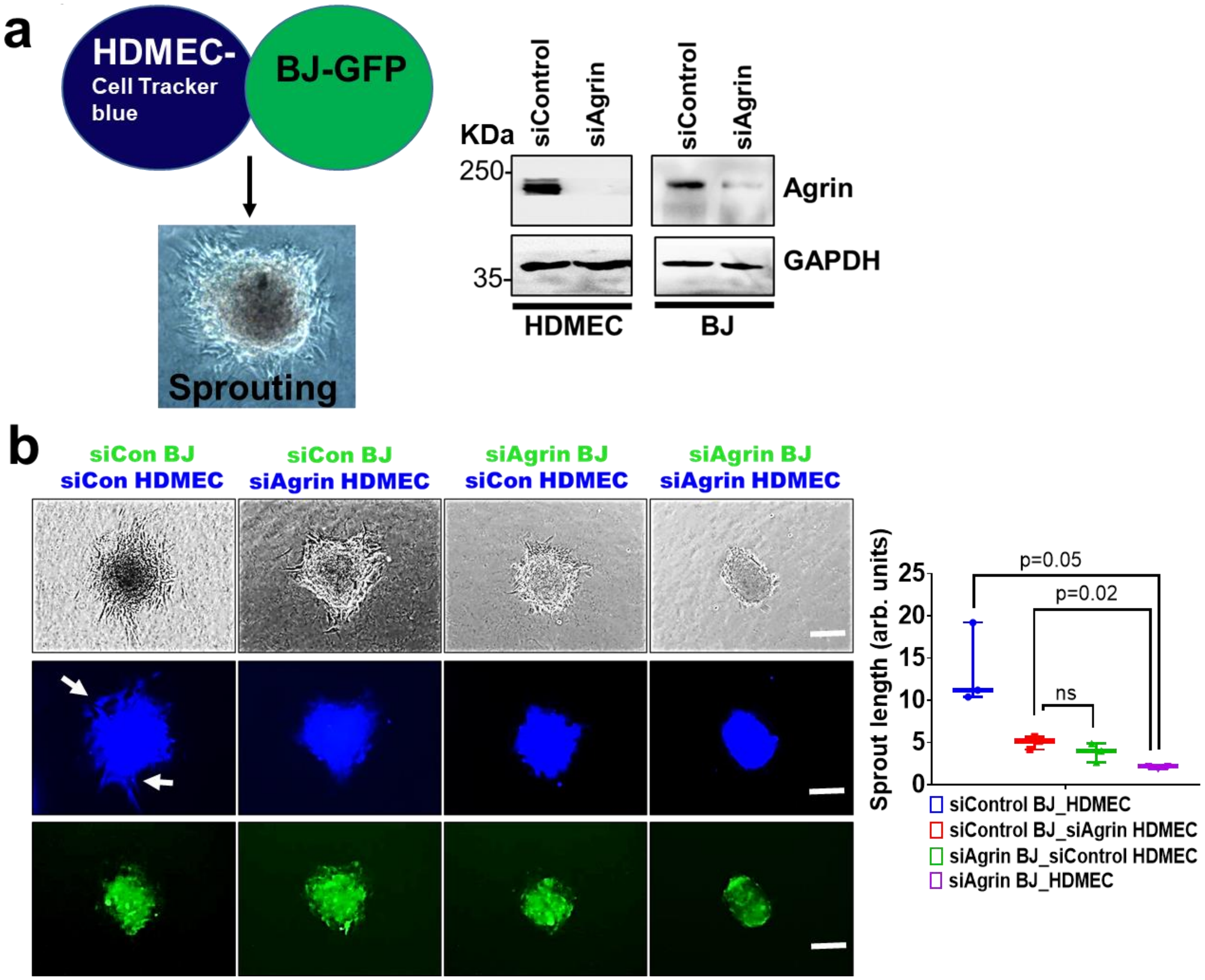

Supplementary Figure 13: Agrin mediates angiogenesis by fostering interactions between dermal fibroblasts and endothelial cells. (a) Western blot analysis in control and Agrin siRNA treated BJ-GFP cells (green) and HDMEC labelled with Cell Tracker blue (blue) for 18h. GAPDH was used as a loading control (n=2 independent experiments). **(b)** Cells treated as in panel (a) were co-cultured to form spheroids for 24h. Bright-field images of sprouting is shown for each indicated condition. The respective fibroblasts and HDMEC spheroids are depicted by green and blue fluorescence, respectively. Scale bar: 100µm. Sprouting was quantified as Box-whiskers plot (n=3 spheroids per condition from three experiments, Whiskers show minimal and maximum values, central line shows median, Two-tailed Students ‘t’ test, *p=0.05 and p=0.02, respectively).

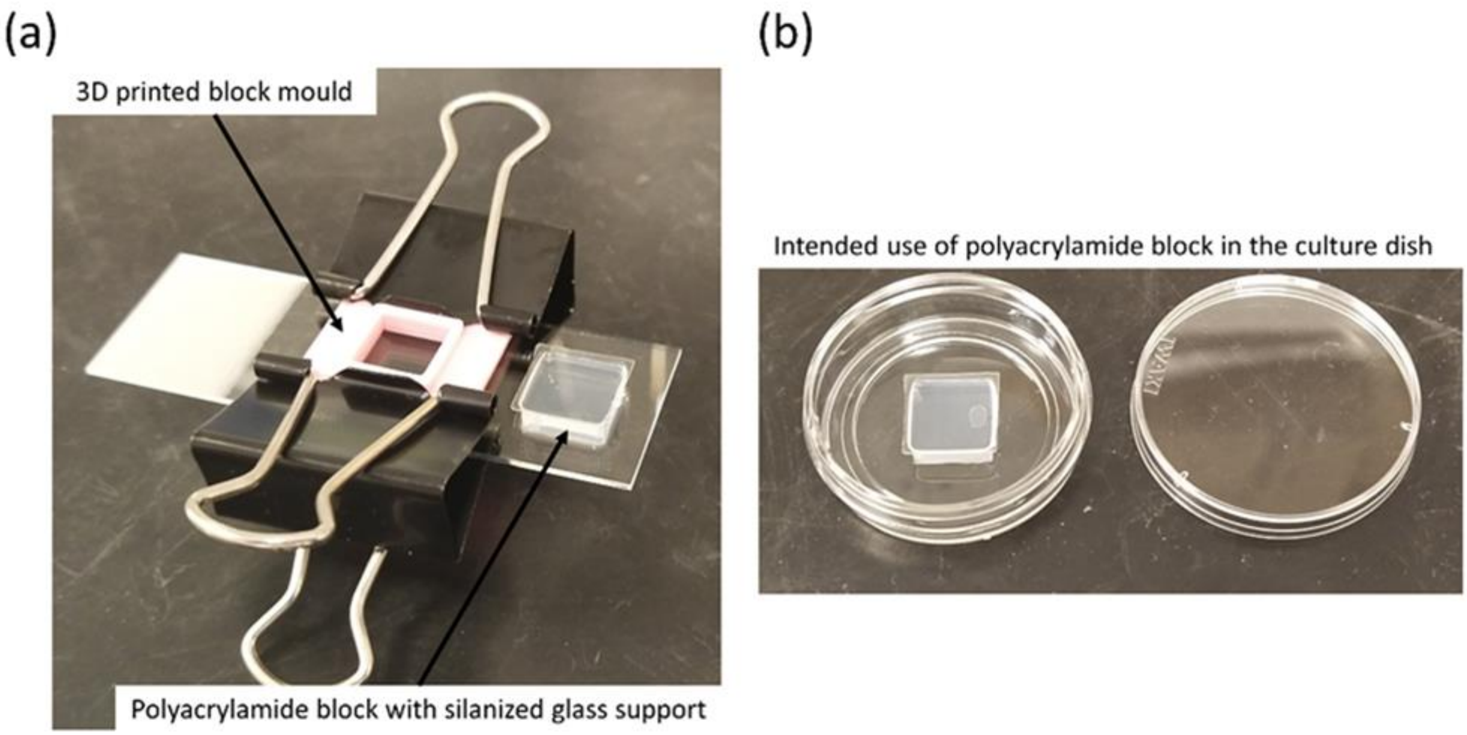

Supplementary Figure 14: Experimental set up for imaging traction force in collectively migrating cells post-wounding. (a) Image showing the 3D-printed mould and casted hydrogel block. **(b)** The image illustrates the intended use of polyacrylamide block on glass bottom dishes to create artificial wounds.

Supplementary Table 1: List of RT-PCR primers used in the study

Name	Sequence	
MMP12	Forward	GATGCACGCACCTCGATGT
	Reverse	GGCCCCCCTGGCATT
MMP10	Forward	GACCCCAGACAAATGTGATCCT
	Reverse	TTCAGGCTCGGGATTCCA
MMP-1	Forward	CCTAGTCTATTCATAGCTAATCAAGAGGATGT
	Reverse	AGTGGAGGAAAGCTGTGCATAC
GSDMB	Forward	AAAGCGACCGGCAATATAAA
	Reverse	ATAGCTCAGGACCCGATTTG
Duox2	Forward	ACGCAGCTCTGTGTCAAAGGT
	Reverse	TGATGAACGAGACTCGACAGC
Dkk3	Forward	GCGGGAGCGAGCAGATCCAG
	Reverse	GGAAGCTGGCAAACTGGCAG
SERPINE2	Forward	TCTCATTGCAAGATCATCGCC
	Reverse	CCCCATGAATAACACAGCACC
SQLE	Forward	TTAGAGGAGAAATGCCAAGGAA
	Reverse	CACTGATGAAGGAGGAAGGAAG
IFI27	Forward	TGGACTCTCCGGATTGACCAAGTT
	Reverse	ATTTGGGATAGTTGGCTCCTCGCT
IFI6	Forward	CCCATCTATCAGCAGGCTCC
	Reverse	AAAGCGATACCGCCTTCTG
ARHGAP26	Forward	TAAGAATGCTTCCAGGACCACTC
	Reverse	GCTGTAACATCTGCCGATTTTTC
TPD52L1	Forward	CTCGGCATGAATCTGATGAATG
	Reverse	TGGCAGTTCCCACGTTATTG
EPHB6	Forward	TGGACTATCAGCTCCGCTACTATG
	Reverse	GTGGCAGTGTTGGTCTCGC
ACAN	Forward	CTACACGCTACACCCTCGAC
	Reverse	ACGTCCTCACACCAGGAAAC
CXCL1	Forward	GCGCCCAAACCGAAGTCATA
	Reverse	ATGGGGGATGCAGGATTGAG
PDK4	Forward	CCGTATTTCTACTCGGATGCTG
	Reverse	TGGCTTGGGTTTCCTGTC
AJAP1	Forward	GGACTCAGCTCCATGTCTATCC
	Reverse	ACTGAGGTCTCCCCTAAGATCC
NPTX4	Forward	GAGAAAGTGGTTGAGAGG
	Reverse	GTAATCAACGACGGCAAG
ACTB	Forward	TGACAGGATGCAGAAGGAGATTA
	Reverse	AGCCACCGATCCACACAGA
GAPDH	Forward	CTATAAATTGAGCCCGCAGC
	Reverse	GACCAAATCCGTTGACTCCG
AGRN	Forward	ACA CCG TCC TCA ACC TGA AG
	Reverse	CCA GGT TGT AGC TCA GTT GC

GPC1	Forward	CGG CCC CGC CAT GGA GCT CC
	Reverse	GGC AGT TAC CGC CAC CGG GG
HSPG2	Forward	AGC ATG GAC GTG GCT GTG CC
	Reverse	GGC GTG CGT GTG TAG CCT GT
**Mouse genes**		
AGRN	Forward	AAT GGC ACG GAC TAA TTT GC
	Reverse	TAT GAG GGT TTG TGG GGT GT
MMP12	Forward	AAT GCT GCA GCC CCA AGG AAT
	Reverse	CTG GGC AAC TGG ACA ACT CAA CTC
GAPDH	Forward	CAT CAC TGC CAC CCA GAA GAC TG
	Reverse	ATG CCA GTG AGC TTC CCG TTC AG

Supplementary Table 2: List of pro-inflammatory cytokines and chemokines primers used in the study

Target Gene (Mouse)	Sequence
IL-18 FORWARD<br><br>IL-18 REVERSE	5′-GCCTCAAACCTTCCAAATCA-3′<br><br>5′-TGGATCCATTTCCTCAAAGG-3′
TGF-BETA 1 FORWARD<br><br>TGF-BETA 1 REVERSE	5’-CCTGTCCAAACTAAGGC-3’<br><br>5’-GGTTTTCTCATAGATGGCG-3’
VEGF FORWARD<br><br>VEGF REVERSE	5’-GTACCTCCACCATGCCAAGT-3’<br><br>5’-TCACATCTGCAAGTACGTTCG-3’
IL1beta FORWARD<br><br>IL1beta REVERSE	5’-CCTTCCAGGATGAGGACATGA -3’<br><br>5’-TGAGTCACAGAGGATGGGCTC -3’
TLR3 FORWARD<br><br>TLR3 REVERSE	5′-CCTCCAACTGTCTACCAGTTCC-3′<br><br>5′-GCCTGGCTAAGTTATTGTGC-3′
MIP-1α FORWARD<br><br>MIP-1α REVERSE	5′-TGAATGCCTGAGAGTCTTGG-3′<br><br>5′-TTGGCAGCAAACAGCTTATC-3′
MIP-1β FORWARD<br><br>MIP-1β REVERSE	5′-TGCTCGTGG CTGCCTTCT-3′<br><br>5′-CAGGAAGTGGGAGGGTCA GA-3′
MIP-2 FORWARD<br><br>MIP-2 REVERSE	5′-CGCCCAGACAGAAGTCATAG-3′<br>5′-TCCTCCTTTCCAGGTCAGTTA-3′
KC FORWARD<br><br>KC REVERSE	5′-TGCACCCAAACCGAAGTCAT-3′<br><br>5′-TTGTCAGAAGCCAGCGTTCAC-3′
TARC FORWARD<br><br>TARC REVERSE	5′-CAAGCTCATCTGTGCAGACC-3′<br>5′-CGCCTGTAGTGCATAAGAGTCC-3′
RANTES FORWARD	5′-CACCACTCCCTGCTGCTT-3′

RANTES REVERSE	5′-ACACTTGGCGGTTCCTTC-3′
MCP-1 FORWARD<br><br>MCP-1 REVERSE	5′-TAGGCTGGAGATCTACAAGAGG-3′<br><br>5′-AGTGCTTGAGGTGGTTGTGG-3′
MIP-3β FORWARD<br><br>MIP-3β REVERSE	5′-AGCCTTCCGCTACCTTCTTA-3′<br><br>5′-GCTGTTGCCTTTGTTCTTGG-3′
I-309 FORWARD<br><br>I-309 REVERSE	5′-CGTGTGGATACAGGATGTTGACAG-3′<br><br>5′-AGGAGGAGCCCATCTTTCTGTAAC-3′
TRIF FORWARD<br><br>TRIF REVERSE	5′-GGTTCACGATCCTGCTCCTGAC-3′<br><br>5′-GCTGGGCCTGAGAACACTCAAG-3′
GAPDH FORWARD<br><br>GAPDH REVERSE	5′-TGAGCAAGAGAGGCCCTATC-3′<br><br>5′-AGGCCCCTCCTGTTATTATG-3′
TNF-α FORWARD<br><br>TNF-α REVERSE	5′-GCCTCTTCTCATTCCTGCTTG-3′<br><br>5′-CTGATGAGAGGGAGGCCATT-3′
IL-6 FORWARD<br><br>IL-6 REVERSE	5′-ACGGCCTTCCCTACTTCACA-3′<br><br>5′-CATTTCCACGATTTCCCAGA-3′
β-actin FORWARD<br><br>β-actin REVERSE	5′-CGTGCGTGACATCAAAGAGAA-3′<br><br>5′-TGGATGCCACAGGATTCCAT-3′

Supplementary Table 3: List of small interfering RNA sequences

Name	Company	Primer Name	Sequence
Human AGRN	Invitrogen (Stealth)	AGRNHSS139721	CCU UUG UCG AGU ACC UCA ACG CUG U
			ACA GCG UUG AGG UAC UCG ACA AAG G
		AGRNHSS180123	CAU ACG GCA ACG AGU GUC AGC UGA A
			UUC AGC UGA CAC UCG UUG CCG UAU G
		AGRNHSS180124	GCG AUU UAU GGA CUU UGA CUG GUU U
			AAA CCA GUC AAA GUC CAU AAA UCG C
Mouse AGRN	Invitrogen (Stealth)	AgrnMSS201833	GGA GAC CUG CCA GUU UAA CUC UGU A
			UAC AGA GUU AAA CUG GCA GGU CUC C
		AgrnMSS201834	AGG UUC CCU UCA GGU GGG CAA UGA A
			UUC AUU GCC CAC CUG AAG GGA ACC U
		AgrnMSS201835	CCA AAG UCC UGU GAU UCC CAG CCU U
			AAG GCU GGG AAU CAC AGG ACU UUG G

Mouse MMP12	Invitrogen (Stealth)	MMP12MSS206678	GGA GCU CAC GGA GAC UUC AAC UAU U
			AAU AGU UGA AGU CUC CGU GAG CUC C

Supplementary methods

Fabrication of polyacrylamide block for collective traction force measurements in a wound healing assay model

A polyacrylamide precursor mixture is first prepared as follows. To prepare 1 ml of such precursor mixture, 200 µl of 40 % w/v aqueous AC (1610140, Bio-Rad), 200 µl of 2 % w/v aqueous BIS (1610142, Bio-Rad), 1.5 µl of TEMED (1610800, Bio-Rad), and 583 µl of Milli-Q water are thoroughly mixed together. 500 µl of this mixture is then combined with 8 µl of 10 % ammonium persulfate (1610700, Bio-Rad) and quickly pipetted into to a 3D printed mould with a cavity size of 1.2x1.2x0.5 cm. A slightly larger square piece of 3-(trimethoxysilyl) propyl methacrylate (440159, Sigma-Aldrich) silanized glass is then placed over the top. The glass will covalently bind to the polyacrylamide once reaction completes, and acts as a solid support for the block; it will also facilitate adding weights to the block. The silanization procedure for this glass support is similar to that performed for the silicone film in the main text, except TMSPMA is used instead of APTES. The solid hydrogel is removed from the mould after 30 minutes and immersed in Milli-Q water overnight to wash out remaining toxic components. The mould and the finished block are shown in Supplementary figure 14a and the intended placement of block in the glass-bottom culture dish is demonstrated in Supplementary Fig. 14b.

Mathematical computation of traction forces

The displacement fields associated with the fluorescent bead images were computed using PIVLab, an open-source Particle Image Velocimetry MATLAB package by Thielicke et al. (2014) [1], which is based on cross-correlation. Images of the substrate in a stress-free state (i.e. sufficiently long after the addition of 1% v/v sodium dodecyl sulfate (L4509, Sigma Aldrich) to the culture to kill the cells) were used as the reference images to compute these deformations. During cross-correlation, four window passes were used, each of (square) window sizes of 64, 32, 16, and 16 pixels respectively, with 50% window overlap between strides. These computed displacement fields were then used to perform Fourier Transform Traction Cytometry (FTTC) to infer traction forces, using the MATLAB code from previous established protocols [2]. The Young's modulus and Poisson's ratio were assumed to be 10 kPa and 0.5 for all of the FTTC calculations, respectively. The L-Curve criterion was used to select the optimal L2 regularisation parameter [3]. More specifically, an L-curve was constructed for the 1st, 10th, 20th, 30th, 40th, 50th, and 60th frame of each sample movie, and then the optimal regularization parameter corresponding to *each L-curve* was selected using the l-corner function in Hasen's (2008) *Regularisation Tools* package for MATLAB [4]. The median of all the optimal regularization (i.e. the parameters calculated for each frame of every sample) was taken to be the optimal regularisation parameter (found to be 1.26×10^{-9}), which was then fixed for all samples. Noting that a larger regularization parameter will reduce the magnitude of our computed force fields, this was done to avoid creating artificial differences in the magnitude of the computed traction fields by systematically selecting smaller or larger regularisation parameters for different sample conditions.

Image Stitching- An in-house script based on image cross-correlation was used to stitch neighbouring image fields together to generate a larger traction force field.

Supplementary references

1. Thielicke, W, Stamhuis, E. PIVlab–towards user-friendly, affordable and accurate digital particle image velocimetry in MATLAB." Journal of open research software 2.1 (2014).
2. Schwarz US, Balaban NQ, Riveline D, Bershadsky A, Geiger B, Safran SA. Calculation of forces at focal adhesions from elastic substrate data: the effect of localized force and the need for regularization. Biophys J, 83(3): 1380-1394 (2002).
3. Kovari DT, Wei W, Chang P, Toro JS, Beach RF, Chambers D, et al. Frustrated Phagocytic Spreading of J774A-1 Macrophages Ends in Myosin II-Dependent Contraction. Biophys J, 111(12): 2698-2710 (2016).
4. Hansen, P C. Regularization tools: a MATLAB package for analysis and solution of discrete ill-posed problems." Numerical algorithms 6.1: 1-35 (1994).
