# Supplementary material for: Agrin-Matrix Metalloproteinase-12 axis confers a mechanically competent microenvironment in skin wound healing
Source: Nat Commun. 2021 Nov 3;12:6349. doi: 10.1038/s41467-021-26717-7 (PMC8566503; doi:10.1038/s41467-021-26717-7)
Supplement: Supplementary file 3 — Description of Additional Supplementary Files [file 41467_2021_26717_MOESM3_ESM.docx]

**Description of additional supplementary files**

**Supplementary data**

**File name**: Supplementary data 1_Upregulated gene list_Agrin Knockdown

**Description**: List of upregulated genes upon Agrin knockdown in HaCaT cells, p values provided by quasi-likelihood (QL) F-test (edgeR).

**File name**: Supplementary data 2_Downregulated gene list_Agrin Knockdown_HaCaT

**Description**: List of down-regulated genes upon Agrin knockdown, p values provided by quasi-likelihood (QL) F-test (edgeR).

**Supplementary movies/videos**

**File name**: Supplementary video 1

**Description**: PIV analysis of collective migration of siControl HaCaT cells

**File name**: Supplementary video 2

**Description**: PIV analysis of collective migration in Agrin depleted HaCaT cells

**File name**: Supplementary video 3

**Description**: PIV analysis of collective migration in Agrin depleted HaCaT cells rescued by adding 10µg/ml sAgrin

**File name**: Supplementary video 4

**Description**: PIV analysis of collective migration of siControl HaCaT cells

**File name**: Supplementary video 5

**Description**: PIV analysis of collective migration in MMP12 depleted HaCaTs

**File name**: Supplementary Movie 6

**Description**: PIV analysis of collective migration in MMP12 depleted HaCaT cells treated with 10µg/ml sAgrin
