# Supplementary material for: Agrin-Matrix Metalloproteinase-12 axis confers a mechanically competent microenvironment in skin wound healing
Source: Nat Commun. 2021 Nov 3;12:6349. doi: 10.1038/s41467-021-26717-7 (PMC8566503; doi:10.1038/s41467-021-26717-7)
Supplement: Supplementary file 12 — Reporting Summary [file 41467_2021_26717_MOESM12_ESM.pdf]

nature portfolio

Corresponding author(s): Sayan Chakraborty and Wanjin Hong

Last updated by author(s): Aug 27, 2021

Reporting Summary

Nature Portfolio wishes to improve the reproducibility of the work that we publish. This form provides structure for consistency and transparency in reporting. For further information on Nature Portfolio policies, see our Editorial Policies and the Editorial Policy Checklist.

Statistics

For all statistical analyses, confirm that the following items are present in the figure legend, table legend, main text, or Methods section.

n/a	Confirmed	
☐	☒	The exact sample size (*n*) for each experimental group/condition, given as a discrete number and unit of measurement
☒	☐	A statement on whether measurements were taken from distinct samples or whether the same sample was measured repeatedly
☐	☒	The statistical test(s) used AND whether they are one- or two-sided<br>*Only common tests should be described solely by name; describe more complex techniques in the Methods section.*
☒	☐	A description of all covariates tested
☒	☐	A description of any assumptions or corrections, such as tests of normality and adjustment for multiple comparisons
☐	☒	A full description of the statistical parameters including central tendency (e.g. means) or other basic estimates (e.g. regression coefficient) AND variation (e.g. standard deviation) or associated estimates of uncertainty (e.g. confidence intervals)
☐	☒	For null hypothesis testing, the test statistic (e.g. *F*, *t*, *r*) with confidence intervals, effect sizes, degrees of freedom and *P* value noted<br>*Give P values as exact values whenever suitable.*
☒	☐	For Bayesian analysis, information on the choice of priors and Markov chain Monte Carlo settings
☒	☐	For hierarchical and complex designs, identification of the appropriate level for tests and full reporting of outcomes
☒	☐	Estimates of effect sizes (e.g. Cohen's *d*, Pearson's *r*), indicating how they were calculated

Our web collection on statistics for biologists contains articles on many of the points above.

Software and code

Policy information about availability of computer code

Data collection	Zen 2 lite (Zeiss), Chemidoc Imaging system Image Lab 6.0.1, Axiovision SE64 Rel 4.9.1, JPK data processing software
Data analysis	GraphPad Prism 7 (https://www.graphpad.com/scientific-software/prism/)<br>Image J 1.52e (https://imagej.nih.gov/ij/)<br>Image Lab 6.0.1<br>Zen 2 lite<br>JPK Version 4.2.1<br>Axiovision SE64 Rel 4.9.1 sp1 (08-2013)<br>MSigDB v 6.2<br>GSEA Software 3 (https://www.gsea-msigdb.org/gsea/index.jsp)<br>Cytoscape 3.4.0

For manuscripts utilizing custom algorithms or software that are central to the research but not yet described in published literature, software must be made available to editors and reviewers. We strongly encourage code deposition in a community repository (e.g. GitHub). See the Nature Portfolio guidelines for submitting code & software for further information.

Data

Policy information about availability of data

All manuscripts must include a data availability statement. This statement should provide the following information, where applicable:

- Accession codes, unique identifiers, or web links for publicly available datasets
- A description of any restrictions on data availability
- For clinical datasets or third party data, please ensure that the statement adheres to our policy

All the data supporting the findings from this study are available within the article and its supplementary information. The RNA sequencing data comparing the gene expression profile of control and Agrin depleted keratinocytes have been deposited on Gene Expression Omnibus (GEO Accession number: GSE179322 https://www.ncbi.nlm.nih.gov/geo/query/acc.cgi?acc=GSE179322).
GSEA (https://www.gsea-msigdb.org/gsea/index.jsp) was performed by Molecular Signatures Database (MSigDB v6.2). Any remaining data will be available from the corresponding authors upon reasonable request. Source data are provided with this paper.

Field-specific reporting

Please select the one below that is the best fit for your research. If you are not sure, read the appropriate sections before making your selection.

[x] Life sciences [ ] Behavioural & social sciences [ ] Ecological, evolutionary & environmental sciences

For a reference copy of the document with all sections, see nature.com/documents/nr-reporting-summary-flat.pdf

Life sciences study design

All studies must disclose on these points even when the disclosure is negative.

Sample size	No prior statistical tests or assumptions were used to determine the sample size of in vitro, ex vivo and in vivo experiments. We chose three biological replicates for in-vitro experiments as it adheres to the commonly held practice in biomedical research, as observed in our previous studies (Chakraborty et. al, Nature Commun, 2015 PMID: 25630468 ; Chakraborty et. al., Cell Reports, 2017 PMID: 28273460 & 2019 PMID: 31340156 , respectively).Our estimated sample sizes are based on what other groups are using in the field. For in-vivo experiments, n defines the number of animal used per condition. For in-vitro experiments, n is equal to biological repeats used. We have mentioned the exact value of n in the respective figure legends.
Data exclusions	No data-points or animals were excluded from the analysis for each experiment.
Replication	Most of our experiments are replicated atleast three times. This information is reflected in the figure legends and the section 'Statistics and reproducibility'. For some experiments, n=2 as indicated in the respective legends. The exact number of technical and/or biological replicates are reflected in the figure legends. Moreover, the results were also replicated in various skin cell types and replicated by different personnel.
Randomization	Animals following punch-biopsy wounding were randomly designated into treatment groups in the concerned experiments. Since our punch-biopsy was of 4mm, we expected similar wound sizes of randomly allocated animal group to begin with. For in-vitro experiments, treatment groups were randomly allocated into each experimental conditions.
Blinding	The in vivo experiments were performed in an non-blinded fashion to comply with the institutional review policy. In some experiments, the animals had to be treated with siRNAs before, so blinded approach will not be applicable for treating these with sAgrin therapy.The in-vitro experiments were also performed in a non-blinded manner. This is because in most cases, the cell types had to be prior-conditioned for knockdown effects and/or sAgrin based treatments. Moreover, the majority of the research investigations were done by multiple personnel to avoid any generic biasness. The results were consistently reproducible by multiple personnel.

Reporting for specific materials, systems and methods

We require information from authors about some types of materials, experimental systems and methods used in many studies. Here, indicate whether each material, system or method listed is relevant to your study. If you are not sure if a list item applies to your research, read the appropriate section before selecting a response.

Materials & experimental systems

n/a	Involved in the study
	[x] Antibodies
	[x] Eukaryotic cell lines
[x]	Palaeontology and archaeology
	[x] Animals and other organisms
[x]	Human research participants
[x]	Clinical data
[x]	Dual use research of concern

Methods

n/a	Involved in the study
[x]	ChIP-seq
[x]	Flow cytometry
[x]	MRI-based neuroimaging

Antibodies

Antibodies used

Mouse monoclonal anti-Agrin (D-2), Santa Cruz Biotechnology, Cat#sc-374117; RRID: AB_10947251 (WB dilution: 1:250, IF-IHC: I:50); Human Agrin clone PIF12 (in house generated, WB; 1:250); Rabbit polyclonal Agrin antibody, Novus Biologicals, Cat# NBP1-90209, IHC 1:50; Mouse monoclonal anti-Integrin β1 Clone P5D2, Abcam, Cat#ab24693; RRID: AB_448230 (IF dilution 1:50); Anti-Integrin Beta1, activated, Clone HUTS-4, Sigma, Cat# MAB2079Z, RRID:AB_2233964 IF dilution 1:50; Mouse monoclonal anti-β Actin (C4), Santa Cruz Biotechnology, Cat#sc-47778; RRID: AB_2714189 WB dilution 1:1000; Rabbit polyclonal anti-GAPDH (FL-335), Santa Cruz Biotechnology, Cat#sc-25778 WB Dilution 1:1000; RRID: AB_10167668; Phospho-Myosin Light Chain 2 (Ser19), Cell Signaling Technologies, Antibody #3671, RRID:AB_330248 IF dilution 1:50, Anti-Myosin light chain (phospho-S20) antibody (ab2480), RRID:AB_303094 WB Dilution 1:1000; Myosin Light Chain 2 Antibody #3672, RRID: AB_330278 WB Dilution 1:1000; MMP-12 Antibody clone G-2, Cat# sc-390863 WB Dilution 1:500; Anti-MMP12 antibody, Abcam, Cat# ab137444 IF-IHC 1:50; MMP12 Polyclonal Antibody, Thermo Fisher Scientific, Cat# PA5-27254, RRID: AB_2544730; Rabbit polyclonal anti-CD31, Abcam, Cat#ab28364; RRID: AB_726362 IF-IHC 1:50; Goat anti-rabbit IgG-HRP, Santa Cruz Biotechnology, Cat#sc-2030; RRID: AB_631747 WB dilution 1:2000; Goat anti-mouse IgG-HRP, Santa Cruz Biotechnology, Cat#sc-2005; RRID: AB_631736; Goat anti-mouse IgM-HRP, Santa Cruz Biotechnology, Cat#sc-2973; RRID: AB_650513 WB dilution 1:2000; Goat anti-Rabbit IgG (H+L) Alexa Flour 488 Invitrogen, Cat#A11034 IF dilution 1:500; Goat anti-mouse IgM Alexa Flour 594, Invitrogen, Cat#A21044 (IF dilution 1:500), Anti-BrdU, Abcam, Cat# ab8152 (IF dilution: 1:50).

Validation

All commercial antibodies have their datasheets published in their respective company websites (this information can be sourced from their catalogue numbers provided above). The commercial antibodies were validated for either Western blotting or Immunofluorescence assays as stated by their company websites and/or previous research outputs. Our results in human cells and mouse tissues were consistent with the available information on the company datasheets and/or previous research works using the same antibodies. We have provided the associated datasheets for easier reference. Please note some antibodies may have been discontinued or the company have changed their catalogues.

Mouse monoclonal anti-Agrin (D-2), Santa Cruz Biotechnology, Cat#sc-374117; RRID: AB_10947251 (WB dilution: 1:250, IF-IHC: I:50); https://www.scbt.com/p/agrin-antibody-d-2

Human Agrin clone PIF12 (in house generated, WB; 1:250);

Rabbit polyclonal Agrin antibody, Novus Biologicals, Cat# NBP1-90209, IHC 1:50; https://www.novusbio.com/products/agrin-antibody_nbp1-90209

Mouse monoclonal anti-Integrin β1 Clone P5D2, Abcam, Cat#ab24693; RRID: AB_448230 (IF dilution 1:50); https://www.abcam.com/integrin-beta-1-antibody-p5d2-ab24693.html

Anti-Integrin Beta1, activated, Clone HUTS-4, Cat# MAB2079Z, RRID:AB_2233964 IF dilution 1:50; https://www.sigmaaldrich.com/SG/en/product/mm/mab2079z?context=product

Mouse monoclonal anti-β Actin (C4), Santa Cruz Biotechnology, Cat#sc-47778; RRID: AB_2714189 WB dilution 1:1000; https://www.scbt.com/p/beta-actin-antibody-c4?requestFrom=search

Rabbit polyclonal anti-GAPDH (FL-335), Santa Cruz Biotechnology, Cat#sc-25778 WB Dilution 1:1000; RRID: AB_10167668; https://www.scbt.com/p/gapdh-antibody-fl-335?requestFrom=search

Phospho-Myosin Light Chain 2 (Ser19), Cell Signaling Technologies, Antibody #3671, RRID:AB_330248 IF dilution 1:50, https://www.cellsignal.com/products/primary-antibodies/phospho-myosin-light-chain-2-ser19-antibody/3671

Anti-Myosin light chain (phospho-S20) antibody (ab2480), RRID:AB_303094 WB Dilution 1:1000;
https://www.abcam.com/myosin-light-chain-phospho-s20-antibody-ab2480.html

Myosin Light Chain 2 Antibody #3672, RRID: AB_330278 WB Dilution 1:1000;
https://www.cellsignal.com/products/primary-antibodies/myosin-light-chain-2-antibody/3672

MMP-12 Antibody clone G-2, Cat# sc-390863 WB Dilution 1:500;
https://www.scbt.com/p/mmp-12-antibody-g-2?requestFrom=search

Anti-MMP12 antibody, Abcam, Cat# ab137444 IF-IHC 1:50;
https://www.abcam.com/mmp12-antibody-ab137444.html

MMP12 Polyclonal Antibody, Thermo Fisher Scientific, Cat# PA5-27254, RRID: AB_2544730;
https://www.thermofisher.com/antibody/product/MMP12-Antibody-Polyclonal/PA5-27254

Rabbit polyclonal anti-CD31, Abcam, Cat#ab28364; RRID: AB_726362 IF-IHC 1:50;
https://www.abcam.com/cd31-antibody-ab28364.html

Goat anti-rabbit IgG-HRP, Santa Cruz Biotechnology, Cat#sc-2030; RRID: AB_631747 WB dilution 1:2000;
https://www.scbt.com/p/goat-anti-rabbit-igg-hrp-cruz-marker-compatible?requestFrom=search

Goat anti-mouse IgG-HRP, Santa Cruz Biotechnology, Cat#sc-2005; RRID: AB_631736;
https://www.scbt.com/p/goat-anti-mouse-igg-hrp?requestFrom=search

Goat anti-mouse IgM-HRP, Santa Cruz Biotechnology, Cat#sc-2973; RRID: AB_650513 WB dilution 1:2000;

Goat anti-Rabbit IgG (H+L) Alexa Flour 488 Invitrogen, Cat#A11034 IF dilution 1:500;
https://www.thermofisher.com/antibody/product/Goat-anti-Rabbit-IgG-H-L-Highly-Cross-Adsorbed-Secondary-Antibody-Polyclonal/A-11034

Goat anti-mouse IgM Alexa Flour 594, Invitrogen, Cat#A21044 (IF dilution 1:500),
https://www.thermofisher.com/antibody/product/Goat-anti-Mouse-IgM-Heavy-chain-Cross-Adsorbed-Secondary-Antibody-Polyclonal/A-21044

Anti-BrdU, Abcam, Cat# ab8152 (IF dilution: 1:50).
https://www.abcam.com/brdu-antibody-iib5-ab8152.html

We have used a custom-made inhouse generated Agrin antibody PIF12 for Western blotting (dilution 1:250). The specificity of this antibody was validated using Agrin siRNA/ KO that showed no Agrin bands and detected human and mouse Agrin. The specificity of MMP12 antibody in Western blot was shown by the reduced band intensity upon treatment with MMP12 specific mouse or human siRNAs. MMP12 antibody also detected the respective protein from human cell lines and mouse tissues.

Eukaryotic cell lines

Policy information about cell lines

Cell line source(s)	Human epidermal keratinocyte cell line HaCaT (Sourced from Thermo Fisher Scientific) was maintained in Dulbecco's modified Eagle's Medium (DMEM) (Gibco) containing 10% fetal bovine serum (FBS) with Penicillin and Streptomycin (Gibco Cat#15140148) antibiotics. The human foreskin normal fibroblasts (BJ) obtained from ATCC (ATCC® CRL-2522™) were passaged in DMEM (Gibco) with antibiotics. Normal primary adult epidermal keratinocytes (HEK) purchased from CELL Applications, Inc., (Cat# C-12003) were maintained in manufacturer provided keratinocyte growth medium. Primary keratinocytes from C57BL mouse strain were purchased from CellBiologics (Cat #C57-6066K) and maintained as per manufacturer recommended Epithelial cell growth medium (Cat#M6621, CellBiologics). The mouse primary dermal fibroblasts were isolated from C57BL mouse strain (Cat#m-GFP-6067), and cultured in complete fibroblast medium (CellBiologics, Cat#M2267).
Authentication	Cell lines were authenticated using ATCC Short-tandem-repeat (STR) profiling cell line authentication services.
Mycoplasma contamination	Cell lines were routinely tested for Mycoplasma every 2-3 months. No Mycoplasma contamination was detected in the experimental cell lines.
Commonly misidentified lines (See ICLAC register)	None

Animals and other organisms

Policy information about studies involving animals; ARRIVE guidelines recommended for reporting animal research

Laboratory animals	We used mouse (Mus musculus) as our laboratory animals.<br>Six to eight to week old female experimental ICR (nomenclature: IcrTac:ICR) mice purchased from InVivos.The mice were housed under standard condition of 21 degrees C with a 12/12 hour light and dark cycle with free access to food. Both male and female mouse pups of ICR strain at day 3 post-birth were used for skin explant assay.
Wild animals	no wild animals were used in the study.
Field-collected samples	no field collected samples were used in the study. The information provided here is not relevant to this field.
Ethics oversight	All wound healing animal experiments were performed in accordance with experimental protocols reviewed by the Biological Resource Center (BRC), Agency for Science Technology and Research (A*STAR) under strict compliance to the Institutional Animal Care and Use Committee (IACUC) guidelines for ethical use of animal models in biological research.

Note that full information on the approval of the study protocol must also be provided in the manuscript.
